# Measurement of thermal properties of white radish (*R*. *raphanistrum*) using easily constructed probes

**DOI:** 10.1371/journal.pone.0171016

**Published:** 2017-03-13

**Authors:** Mfrekemfon Samuel Obot, Changcheng Li, Ting Fang, Jinquan Chen

**Affiliations:** 1 College of Food Science, Fujian Agriculture and Forestry University, Fuzhou, Fujian, China; 2 Department of Agricultural and Food Engineering, Faculty of Engineering, University of Uyo, Uyo, Akwa Ibom State, Nigeria; North China Electric Power University, CHINA

## Abstract

Thermal properties are necessary for the design and control of processes and storage facilities of food materials. This study proposes the measurement of thermal properties using easily constructed probes with specific heat capacity calculated, as opposed to the use of Differential Scanning Calorimeter (DSC) or other. These probes were constructed and used to measure thermal properties of white radish in the temperature range of 80–20°C and moisture content of 91–6.1% wb. Results showed thermal properties were within the range of 0.71–0.111 Wm^-1^ C^-1^ for thermal conductivity, 1.869×10^−7^–0.72×10^−8^ m^2^s^-1^ for thermal diffusivity and 4.316–1.977 kJ kg^-1^C^-1^for specific heat capacity. These results agree with reports for similar products studied using DSC and commercially available line heat source probes. Empirical models were developed for each property through linear multiple regressions. The data generated would be useful in modeling and control of its processing and equipment design.

## Introduction

Thermal properties are those physical properties of a material that are significant in heat transfer problems[1]. Some of these properties include: dimensional characteristics, density, fluid viscosity, unit surface conductance latent heat, specific heat, thermal conductivity, thermal emissivity, thermal diffusivity, coefficient of thermal expansion etc. The dependence of these properties on the temperature of the materials’ ambience and their moisture content is necessary in the modeling, simulation and equipment design of various food processing operations [2]. Process thermal controls can only be put to use by the precise knowledge of the thermal properties of the food. The primary thermal properties are specific heat capacity (at constant pressure), thermal conductivity and thermal diffusivity. The specific heat energy measures the amount of energy that is required to raise the temperature of a body by 1°C; thermal conductivity is an intrinsic property which measures the ability of a substance to conduct heat, while thermal diffusivity is the ability of a material to conduct thermal energy relative to its ability to store energy. To calculate heat transfer in a food, its thermal properties, geometry and thermal processing condition are used as the major parameters [3].

Compositional model is used when there are no empirical data for the agricultural product. It calculates the desired parameter based on the composition of product as proposed by an early researcher. According to [4], it is not always accurate to use the general compositional model to predict thermal properties of each specific food material based on its compositions and temperature because this assumes that each component has the same thermal properties regardless of structure in different food materials.

Differential scanning calorimeter and KD2 pro (thermal properties analyser produced by Decagon devices) are the equipments that are mostly used in the studies of thermal properties of material but due to their high cost, they are not always available. In view of this, [5] had proposed a new device to measure the thermal property of polystyrene and compared his results with that produced by the differential scanning calorimeter. The equipment according to the author is easy to construct. However the thermal properties of high moisture biomaterials may not be easy to be measured using the equipment proposed by[5]. The equation connecting the primary thermal properties is:

$$\alpha =\frac{k}{\rho C_{p}}$$ 1

where α: thermal diffusivity; k: thermal conductivity; ρ: density; C_p_: specific heat capacity.

White radish is an important vegetable in many countries. White radish belongs to the Cruciferae or mustard family and is a biennial. This root is getting so important. Apart from its culinary uses [6], has it that radish root contains some coumarins, enzymes, organic acids, phenolic compounds and some sulphuric compounds. To the knowledge of the authors of this work, there is no data for the thermal properties of white radish in the literature. Therefore, this study demonstrates the measurement of thermal properties using the inexpensive thermal property (line heat source) probes in the temperature range of 80–20°C and moisture content of 91–6.1% wb. The constructed probes were able to generate data that could be compared to that of similar crops in the literature. The data and the models obtained from this study will be useful for the storage, process design and control of this economically important vegetable.

## Materials and methods

### Materials

The white radish roots used for this experiment were bought from a local store in Fuzhou city near to the east gate of Fujian Agriculture and Forestry University, **26°5′16″N 119°14′6″E**, Fujian Province P R China. The radish roots had an initial moisture content of 91% wet basis as determined by drying a known weight in a hot air oven for 24 hrs at 105°C. Fresh radish roots were cut into same size and volume by using a stainless steel cylinder to bore the root (this was done for uniformity of conditioning results), these were conditioned to desired moisture content by drying in a hot air oven at 70°C for 0 hr, 2 hrs, 5 hrs and 9.5 hrs which corresponded to 91, 64, 31 and 6.1% moisture content (wb) respectively. They were further grated using a hand held grater and filled into the sample holder for further analysis. The bulk density was measured as mass/volume after filling into the sample holder

#### Thermal conductivity probe

**Fig 1:**
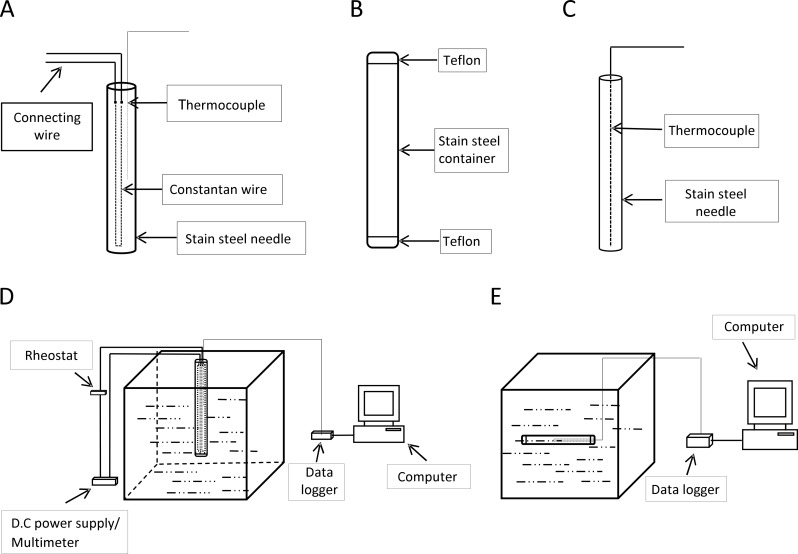
The thermal property probes apparatus and their experimental setup. a) Thermal conductivity probe. b) Cylindrical sample container. c) Thermal diffusivity probe. d) Experimental set up for the thermal conductivity. e) Experimental setup for the thermal diffusivity.

A line heat source probe was used to measure thermal conductivity. It was designed for this study following the description of [2]. The probe was made of a stainless steel needle containing a T -type thermocouple and a constantan heating wire as shown in Fig 1A, the stainless steel needle was 110 mm long and 2.02 mm in diameter. The heating wire (0.47 mm diameterand 200 mm long) and the thermocouple (0.64 mm diameter and 55 mm long) were inserted into the needle. The heating wire was bent into two equal parts length wise with its ends joint to connecting wires thus having its entire length within the probe. It was calibrated using 0.5% agar solution, glycerin and powdered milk at 30°C.

The cylindrical sample container (Fig 1B) was a stainless steel tube of 150 mm long, 28 mm diameter and 1.5 mm thick. Both ends of the tube were covered with Teflon material- one end was permanently sealed while the other end was temporary sealed after filling in the sample.

#### Thermal diffusivity probe

The thermal diffusivity probe designed for this study was made of a stainless steel needle containing a T-type thermocouple as shown in Fig 1C. The needle was 0.6 mm in diameter and 70 mm long. The length of the thermocouple within the needle was as 69 mm and connected to a data logger. The probe was calibrated using water according to the method described by [7]: The sample container was filled with water of at 60°C with the thermal diffusivity probe inserted and placed in a thermostatic water bath containing water at 10°C and the time-temperature data was logged with a data logger.

#### Data logger

The data logging was done using Tracer DAQ (USB 200 series) of Measurement Computing Corporation USA, connected to a computer.

### Methods

#### Thermal diffusivity

The prepared material was tightly filled into cylindrical sample container and then covered. The thermal diffusivity probe was then inserted length wise at the center of the tube through the Teflon cover and then the hole through which the needle passed was sealed with a water resistant sealant. This assembly was put into the thermostatic water bath at 80, 60 or 40°C. The temperature time graph of both the core of the tube (sample) and the water bath were logged by the data logger until the sample temperature equated the set water bath temperature. Schematic diagram of the setup was as shown in Fig 1E.

Thermal diffusivity was determined using the 1D Fourier equation applied to a cylinder. It has a high precision and does not depend on the exact place which the temperature is measured but has high accuracy when measured at the core of the cylinder [7]. A graph of *ln*Θ = *f*(*t*) was drawn The dimensionless temperature, Θ was calculated from Eq 2. And the thermal diffusivity was calculated from the curve according to [7] using Eq 3. Each experiment was done four times and the mean value was reported.

$$\Theta =\frac{T_{c}-T_{b}}{T_{0}-T_{b}}$$ 2

Where T_c_, T_b_ and T_0_ are temperature at the core of the sample container, temperature of the water bath and initial temperature of the sample respectively, all in °C.

$$\alpha =\frac{1}{\tau }{\left(\frac{r}{2.045}\right)}^{2}$$ 3

Where α = thermal diffusivity, m^2^ s^-1^, τ = time constant, r = radius of the sample container, m

#### Prediction equation for thermal diffusivity

The Marten’s equation (Eq 4) was used for the prediction of the thermal diffusivity. Its prediction was compared to the prediction of the empirical equation of this study and the experimental data. This equation was appropriate because it includes the addition of temperature to moisture content for the prediction of thermal diffusivity.

$$\alpha =\left(0.057363M+0.000288\left(T+273\right)\right)\times 10^{-6}$$ 4

where α = bulk thermal diffusivity (m^2^s^-1^), T = temperature (°C) and M = moisture content (g water/g product)

#### Thermal conductivity

The experiment was done by inserting the thermal conductivity probe into the center of the cylindrical sample container which was tightly filled with prepared sample. The prepared samples in the container were equilibrated in a thermostatic water bath at temperatures of 40, 60 or 80°C, depending on the experiment. A digital multimeter (Zhaoxin RXN-303D, China) which also acted as a D.C power supply was preset to 1.51 A and 8.3 V by the use of a sliding rheostat. The setup was as shown in Fig 1C. When the sample temperature reached equilibrium with the water temperature, the heater wire was energized using the regulated DC power supply consisting of 1.51 A and 8.3 V (and maintained by the variable resistor (rheostat) during the experiment) to allow a heat transfer from the probe to the surrounding sample in a radial fashion. The resulting temperature rise was measured using the thermocouple located in the probe (T type thermocouple) and recorded every second over the course of a 5 min period using the data logger [2,8]. A graph of T (temperature) versus ln (t) (time) was plotted and the thermal conductivity was calculated using Eq 5. Each experiment had at least 5 replicates.

$$k=\frac{Q}{4\pi S}$$ 5

Where Q = Heat input = I^2^×R, I = current (A) and R = resistance (Ω), k = thermal conductivity (Wm^-1^°C^-1^), S = slope of the straight portion of the graph

The prediction equation used for the thermal conductivity was the one proposed by [9] for the thermal conductivity, its prediction was compared to that predicted by the experimental data and the empirical equation of this study.

#### Specific heat capacity

The specific heat capacity was calculated using Eq 1.The prediction equation used for the thermal conductivity was the one proposed by [9] for the specific heat. Its prediction was compared to the experimental data and that predicted by the empirical equation of this study.

### Experimental design

The experiments were conducted at four levels of moisture content (6.1%, 31%, 64% and 91% wb) and 4 levels of temperature (20, 40, 60, and 80°C). Each experiment was done at least four times and the average value was used for analysis. Linear multiple regressions were carried out at 95% confidence interval to determine the effects and significance of each parameter for each thermal property studied using SPSS 16

## Results and discussions

The moisture content at fresh weight was 91% wet basis. The results of the thermal conductivity probe calibration were 0.630 (±0.012) Wm^-1^°C^-1^ for 5% agar gel, 0.0653 (±0.001) Wm^-1^°C^-1^ for powdered milk respectively. These were compared to the values published by [10] which were 0.628 Wm^-1^°C^-1^ and 0.0650 Wm^-1^°C^-1^ for 5% agar gel and powdered milk respectively at 30°C.

### Thermal conductivity

**Fig 2:**
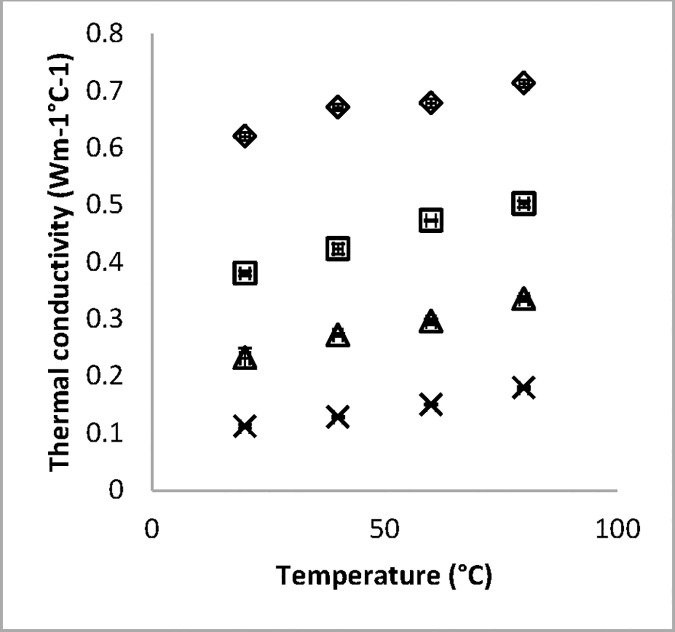
The experimental data (with error bars) showing the variation of thermal conductivity of radish. Temperature range of 20 and 80°C and moisture content of Δ 91, ◊ 64, □ 31, and × 6.1% moisture content (wb).

The bulk thermal conductivity of white radish for moisture content of 91% to 6.1% moisture content and at temperature of 80–20°C was found to vary from 0.71 to 0.111 W m^-1^°C^-1^. Fig 2 shows the thermal conductivity of white radish and its variation with both moisture and temperature. For each level of moisture studied, the thermal conductivity of white radish increased with increase in temperature, at a given temperature the thermal conductivity also increased with increase in moisture content.

Both factors were found to be highly significant after being correlated using linear multiple regression at 95% confidence interval. The effect of moisture on thermal conductivity of radish was higher than that of temperature and more significant. At Fresh weight (91% moisture content), effect of temperature at the range studied (80–20°C) was 0.09 Wm^-1^°C^-1^. However, at subsequent moisture content of 64, 31 and 6.1%, the effects of temperature changed to 0.12, 0.11 and 0.069 Wm^-1^°C^-1^ respectively. Higher moisture content exhibited higher thermal conductivity possibly because of the higher the number of ions and dipoles. High temperatures therefore caused high gyration of available ions and dipoles enabling faster heat transfer [11]. At lower moisture there are fewer ions and dipoles leading to slower heat transfer. This may explain the high effects of moisture content over temperature. The relationship between the temperature and moisture content of radish on the thermal conductivity was done using a multiple regression correlation (Eq 6).

$$k=0.006+2\times 10^{-3}T+0.61M$$ 6

Table 1 shows the empirical equation for this relationship and its statistical information where T and M respectively represent temperature and moisture content within the range of this study. The high R^2^ and a low standard error of estimate are as a result of a good agreement between the model and measured data. It is worth mentioning that the values of k from this study was within the range reported in the literature of high moisture agricultural product: 0.668 Wm^-1^°C^-1^ for straw mushroom [4]; 0.57 Wm^-1^°C^-1^ for cassava, 70% wb at 30°C [12]; 0.70 for spinach [13]; 0.60 for yam, 79% wb at 24.8°C. [11,14] also reported thermal conductivity as low as in this this study for lower moisture content and using the same method.

**Table 1:** Regression empirical equation for the thermal conductivity of white radish and its statistical information.

Empirical model	F-value	P-value	R^2^	SEE
$k=0.006+2\times 10^{-3}T+0.61M$	405.04	0.00	0.984	0.074

**Fig 3:**
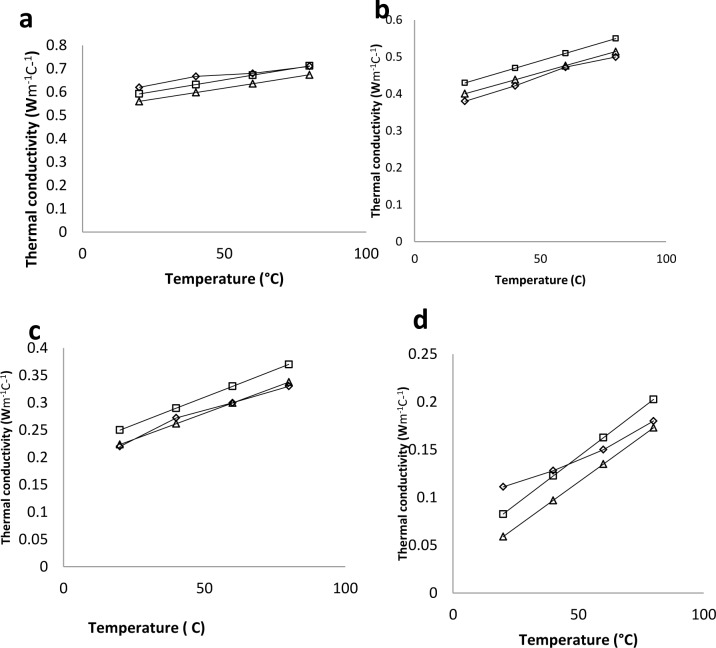
Experimental data and predicted thermal conductivity of white radish in temperature range of 80–20°C. a) 91% wb (b) 64% wb (c) 31% wb (d) 6.1% wb. ◊ = Experimental data. □ = Empirical equation from Table 1. $k=0.006+2\times 10^{-3}T+0.61M$ (R^2^ = 0.984) (0.061<M<0.91, 20<T(C) < 80). Δ = Vagenas et al, (1990) equation k=0.015+0.0019T+0.59M. (R^2^ = 0.980) (0.3<M<0.95, 0<T(C) <90).

The [9] model for thermal conductivity (Eq 7) was used to estimate and to compare its estimations to that of the empirical model and the experimental data from this study (Fig 3) where the empirical equation for thermal conductivity of white radish was Eq 6 and compared to that of [9]. It was observed that both models followed the same trend.

k=0.015+0.0019T+0.59M 7

### Thermal diffusivity

**Fig 4:**
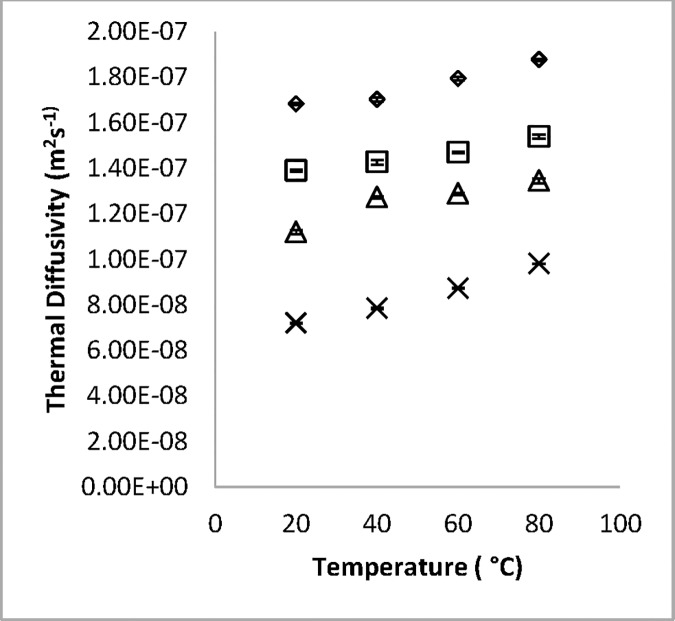
Observed variation of thermal diffusivity of white radish roots with temperature (with error bars). Temperature range of 20 and 80°C and moisture content of Δ 91, ◊ 64, and × 6.1% moisture content (w.b).

Bulk thermal diffusivity is mostly calculated in reported literature from thermal conductivity, density and specific heat capacity. The equipment used in measuring the specific heat capacity is usually expensive (such as the differential scanning calorimeter), hence a probe was designed to measure the thermal diffusivity in this study. The probe used was calibrated by measuring the thermal diffusivity of water as it cooled from 60 to 10°C which yielded 1.482×10^−7^ m^2^s^-1^. The value in the reference article [7] was 1.498×10^−7^ m^2^s^-1^which resulted in 1.0 percent difference. The maximum value for the diffusivity of white radish in this study was 1.869×10^−7^ m^2^s^-1^ which was the value for the fresh weight at 80°C while the lowest value was 0.72×10^−8^ m^2^s^-1^ at 6.1% moisture at 20°C. The results of the thermal diffusivity of white radish roots are shown on Fig 4. The results showed that increase in temperature made the radish roots at particular moisture content be more diffusive. Thermal diffusivity increased linearly with the increase in temperature and moisture. This is in agreement with reported research on agricultural products. A multiple regression was carried out to correlate the effects of moisture and temperature on the thermal diffusivity of white radish (Eq 8). The empirical model and its statistical information are on Table 2, where T and M respectively are temperature and moisture content within the range of this study.

$$\alpha =\left(0.669+3\times 10^{-3}T+1.02M\right)\times 10^{-7}$$ 8

**Table 2:** Regression empirical equation for the thermal diffusivity of white radish and its statistical information.

Empirical model	F-value	P-value	R^2^	SEE
*α* = (0.669 + 3 × 10^−3^*T* + 1.02*M*) × 10^−7^	165.45	0.00	0.967	0.074

**Fig 5:**
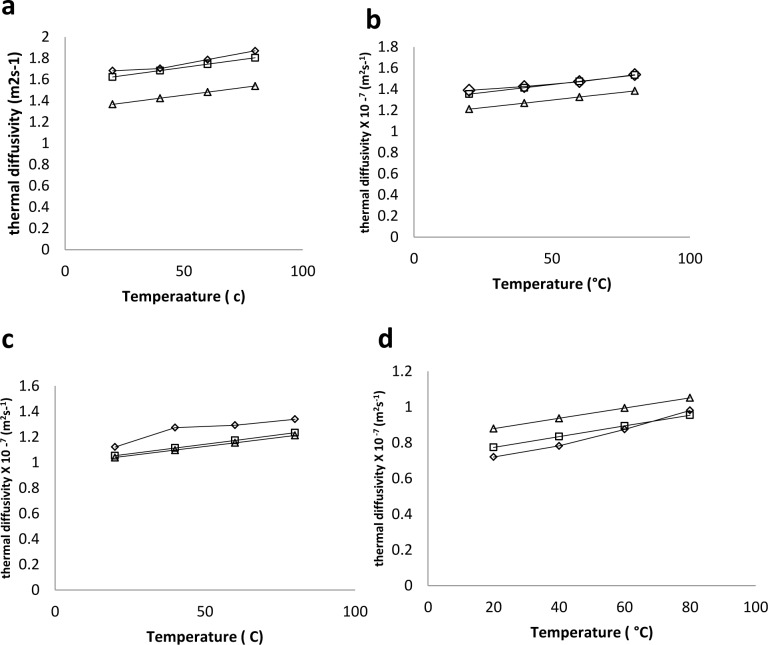
Experimental data and predicted thermal diffusivity of white radish. a) 91% wb (b) 64% wb (c) 31% wb (d) 6.1% wb. ◊ = Experimented data. □ = Empirical equation from Table 2. *α* = (0.669 + 3 × 10^−3^*T* + 0.1*M*) × 10^−7^ (R^2^ = 0.967) (0.061<M<0.91, 20<T (°C) < 80). Δ = Martens equation *α* = 0.057363*M* + 0.000288(*T* + 273) × 10^−6^ (R^2^ = 0.9512).

The high F-value, R^2^ and low Standard error of estimate indicate a good fit of this model to the experimental values. The effects of the two factors were seen to be significant at the confidence interval of 95% but as in the case of thermal conductivity, the effect of moisture is more significant as shown by their coefficients in the empirical equation. This is in agreement with earlier researches by [4,15,16]. Other researchers also reported increase of thermal diffusivity with increasing moisture content [12,17,18]. Marten’s equation (Eq 9) was used as the prediction model for the thermal diffusivity of white radish. Fig 5 compares the Martens model (Eq 9), the empirical model (Eq 8) and the experimental data. The R^2^ for the two models are very close in terms of values

$$\alpha =0.057363M+0.000288\left(T+273\right)\times 10^{-6}$$ 9

### Specific heat capacity

The specific heat capacity (at constant pressure) as the amount of heat needed to raise the temperature of a substance by 1°C was calculated from Eq 10 which is a rearranged form of Eq 1.

$$c_{p}=\frac{k}{\rho \alpha }$$ 10

where Cp is the specific heat capacity (kJ kg^-1^°C^-1^), ρ is the density (kgm^-3^), k = thermal conductivity

**Fig 6:**
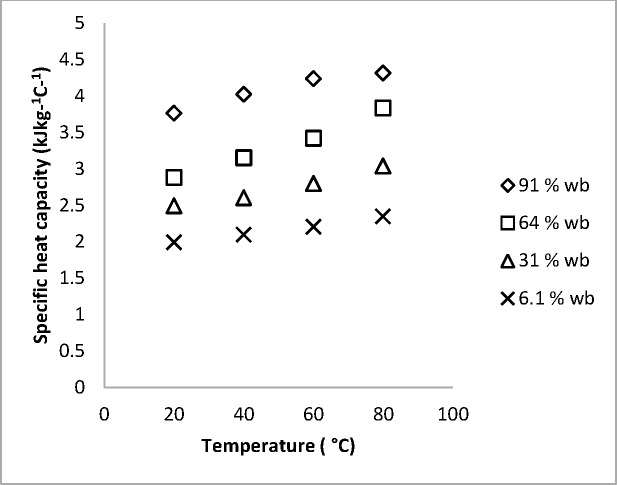
Observed variation of Specific heat capacity of white radish roots. Temperature range of 20 and 80°C and moisture content of Δ 91, ◊ 64, □ 31 and × 6.1% moisture content (w.b).

The bulk density ranged between 980 and 780.2 kg m^-3^. The specific heat capacity of white radish in this study was between 4.316 and 1.977 kJ kg^-1^°C^-1^. The value of specific heat for the product at fresh weight (91% wb) was in the range of values reported in the literature for agricultural products of about the same moisture content and temperature. The specific heat capacity of straw mushroom at 90% (wb) was reported as 4.008 kJ kg^-1^ C^-1^ [4], while that of spinach was 4.3 kJ kg^-1^ C^-1^, [13]. The reported values in the literature though measured using the differential scanning Calorimeter (DSC), are in agreement with our results. The specific heat capacity of radish like the thermal diffusivity and the thermal conductivity increased with the increase in temperature for each level of moisture content studied (Fig 6). The correlation between the moisture content and temperature shows that the two factors were highly significant with moisture content being more significant than temperature. The empirical model, Eq 11 is shown on Table 3 (with its statistical information) where T and M respectively are temperature and moisture content within the range of this study.

$$C_{p}=1.472+0.011T+2.22M$$ 11

**Table 3:** Regression empirical equation for the bulk specific heat of white radish and its statistical information.

Regression equation	F value	P value	R^2^	SEE
*C*_*p*_ = 1.472 + 1.1 × 10^−2^*T* + 2.22*M*	345.06	0.00	0.982	0.1143

**Fig 7:**
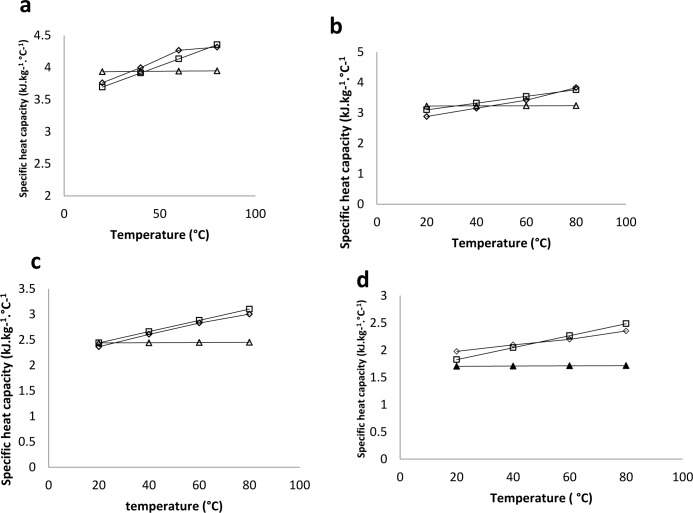
Experimental data and predicted specific heat capacity of white radish. a) 91% w. b (b) 64% wb (c) 31% wb (d) 6.1% wb. ◊ = Experimental data. □ = Empirical equation from Table 3. *C*_*p*_ = 1.472 + 0.011*T* + 2.22*M* (R^2^ = 0.982) (0.061<M<0.91, 20<T (°C) < 80). Δ = Vagenas et al, (1990) equation *C*_*p*_ = 1.54 + 2.03 × 10^−4^*T* + 2.627*M* (R^2^ = 0.9612) (0.3<M<0.95, 0<T (°C) <90).

The high R^2^, high F- value and low standard error of the estimate indicate the goodness of fit. The model used for the estimation of the specific heat capacity of white radish was the[9] model for specific heat capacity(Eq 12). Fig 7 shows the estimations of [9] (Eq 12), empirical model (Table 3) and the experimental data. The R^2^ for the two models were above 0.9.

$$C_{p}=1.54+2.03\times 10^{-4}T+2.627M$$ 12

## Conclusion

The thermal properties of white radish were measured using easily constructed line heat source probe and the thermal diffusivity probe in this study and the effects of temperature and moisture on white radish were evaluated within the range of 20–80°C and 91–6.1% wb respectively. The thermal conductivity was between 0.71 and 0.111 W m^-1^°C^-1^, the thermal diffusivity was between 1.869×10^−7^ m^2^s^-1^ and 0.72×10^−8^ m^2^s^-1^ and the specific heat was between 4.316 and 1.977 kJ kg^-1^°C^-1^.Our results were in agreement with earlier studies in terms of range of values and in the trend of results. The results of this study were fitted to appropriate thermal property models in each case. They all had good fits showing the reliability of the measured data. The specific heat capacity was calculated from the values measured with the thermal conductivity and diffusivity probes. Both thermal diffusivity and specific heat were in the range reported in the literature for materials with similar properties. The regression models from this study may be used to control storage and processing of white radish.

The data generated is very important and can be used in proper processing of white radish
